# Forecasting Waitlist Trajectories for Patients With Metabolic Dysfunction–Associated Steatohepatitis Cirrhosis: A Neural Network Competing Risk Analysis

**DOI:** 10.2196/68247

**Published:** 2026-01-29

**Authors:** Gopika Punchhi, Yingji Sun, Eunice Tan, Naomi Khaing Than Hlaing, Chang Liu, Sumeet Asrani, Sirisha Rambhatla, Mamatha Bhat

**Affiliations:** 1 Schulich School of Medicine and Dentistry, Western University, London, ON, Canada; 2 Ajmera Transplant Centre, University Health Network, 585 University Ave, Toronto, ON, M5G 2N2, Canada, 1 416-581-7512; 3 Division of Gastroenterology, Department of Medicine, National University Hospital, Singapore, Singapore; 4 Department of Medicine, Yong Loo Lin School of Medicine, National University of Singapore, Singapore, Singapore; 5 Systems Design Engineering, University of Waterloo, Waterloo, ON, Canada; 6 Division of Hepatology, Baylor University Medical Center, Dallas, TX, United States; 7 Department of Management Sciences, University of Waterloo, Waterloo, ON, Canada; 8 Division of Gastroenterology and Hepatology, University of Toronto, Toronto, ON, Canada

**Keywords:** liver transplantation, metabolic dysfunction-associated steatohepatitis, waitlist trajectory, metabolic dysfunction, liver disease, cirrhosis, neural network, risk analysis, hepatic Disease, predictive, prediction, deep learning, liver transplant

## Abstract

**Background:**

Metabolic dysfunction–associated steatohepatitis (MASH) cirrhosis is a leading indication for liver transplantation (LT). Patients with MASH cirrhosis are complex and often have extensive comorbidities. The current model for end-stage liver disease (MELD)–based liver allocation system has suboptimal concordance in predicting waitlist mortality for patients with MASH cirrhosis. Furthermore, it does not capture the competing outcomes of death and LT on the liver transplant waitlist.

**Objective:**

A competing risk analysis using deep learning was conducted to forecast waitlist trajectories of patients with MASH cirrhosis using data available at the time of waitlisting.

**Methods:**

A deep learning competing risk model was constructed using data from 17,551 waitlisted patients with MASH cirrhosis in the Scientific Registry of Transplant Recipients (SRTR) based on the DeepHit model framework with five-fold cross-validation. Model performance was evaluated and compared to single-risk Cox proportional hazards and random survival forests (RSF) models in predicting death or transplant using the concordance index and Brier score. Additionally, a novel performance metric, the competing event coherence (CEC) score, was developed to evaluate model performance in the setting of competing risks. Features associated with death and transplant in the DeepHit model were identified using permutation importance. Models were externally validated on data from the University Health Network.

**Results:**

A total of 17,551 patients were included. The mean MELD at listing was 19.4 (SD 8.1). At 120 months of follow-up on the waitlist, 54.6% (9599/17551) of patients underwent LT, 25.6% (4510/17551) of patients died or were removed due to deterioration, and 19.8% (3442/17551) of patients were removed for improvement or were censored. In a competing risk scenario, DeepHit achieved the best CEC scores at 1 (0.813), 3 (0.811), 6 (0.794), and 12 months (0.772) on the waitlist. The cause-specific RSF model had the highest concordance indices for death or transplant at all time points (death: 0.874 at 1 month, 0.840 at 6 months, and 0.814 at 12 months) except for death at 3 months, where DeepHit (0.883) outperformed RSF. RSF also had lower Brier scores overall, except for transplant at 12 months, where DeepHit outperformed RSF (0.206 vs 0.228). These results were similar on external validation. On feature importance assessment, MELD at listing and its components, as well as functional status, age, and blood type, were associated with death and transplant on the waitlist.

**Conclusions:**

A deep learning competing risk analysis can forecast the risks of both death and transplant in patients with MASH on the waitlist, helping to inform clinical decisions by identifying the most impactful covariates for each outcome.

## Introduction

Metabolic dysfunction-associated steatohepatitis (MASH) cirrhosis is a leading cause of liver transplantation (LT) globally and the fastest growing indication for LT in the United States, with the prevalence of waitlisted candidates increasing from 2.5% to 20.4% between 2004 and 2019. The prevalence of MASH cirrhosis is projected to continue to rise significantly in the coming years [1,2]. Candidates waitlisted for LT are prioritized based on their model for end-stage liver disease (MELD) score, which has been periodically reviewed and updated, most recently to the MELD 3.0 score [3]. The MELD-based scoring system predicts waitlist mortality and does not account for type of liver disease, as previous studies have demonstrated minimal effects on the predictive performance [4]. Despite changes to MELD-based scoring systems in recent years and the increasing prevalence of MASH, MELD-based scoring systems have lower concordance in candidates with MASH cirrhosis compared to those listed with other liver diseases, highlighting the need to develop waitlist prediction models that capture the complexity of MASH cirrhosis [3-6]. There are several possible explanations for the lower concordance of MELD models in this population. Patients with MASH, particularly those in the low- to mid-MELD score range, tend to have faster disease progression than is captured by their MELD score progression, higher pre-LT mortality risk, and lower likelihood of recovery than other patients on the waitlist. Additionally, patients with MASH cirrhosis tend to develop clinically significant portal hypertension at lower MELD-sodium (MELD-Na) scores, contributing to higher waitlist mortality [5-8]. Despite having more severe comorbidities, such as portal hypertension, advanced age, higher BMI, diabetes, hypertension, and hyperlipidemia, patients with MASH cirrhosis are less likely to receive a transplant on the waitlist and more likely to face higher waitlist mortality and removal due to becoming too ill to undergo transplant compared to patients listed with other liver diseases [8-12]. Improving waitlist prediction in patients with MASH through developing MASH-specific models can aid clinicians in optimizing their waitlist outcomes and pretransplant status, thus potentially improving overall waitlist outcomes.

Another limitation of MELD-based models is that they provide information on the risk of death on the waitlist without accounting for the risk of transplantation, censoring patients who do not experience the event of interest and leading to biased estimation of risk [7]. These single-risk Cox proportional hazards (CoxPH)–based models cannot predict risk while accounting for the possibility that a patient can experience multiple events at a given time point on the waitlist, while a censored patient can experience a competing event that would make the primary event of interest clinically impossible (ie, a patient who undergoes LT cannot also die on the waitlist). The risks of each event cannot be compared between multiple cause–specific models. In contrast, competing risk models account for multiple mutually exclusive events [13]. Compared to traditional regression methods, machine learning (ML) can handle large, heterogeneous datasets and avoid several fundamental assumptions of linearity and proportionality that CoxPH models make [14-16]. For example, DeepHit is a deep learning competing risk neural network model that captures intricate, nonlinear interactions between several factors and outcomes (such as mortality and transplant) in one model [9,11,17,18]. By using a DeepHit-based model to predict waitlist outcomes, clinicians will better understand the trajectory of patients with MASH cirrhosis with numerous comorbidities who are at high risk of both mortality and transplantation. Patients with MASH at lower risk of death and transplant may be better candidates for living donor liver transplantation (LDLT) and can be redirected accordingly [19]. Due to the fundamental differences between competing risk and single-risk settings, current model evaluation metrics, such as the concordance index (C-index) and Brier score, may be inadequate for use in competing risk settings [12,20]. We design and propose the competing event coherence (CEC) score, a novel performance metric to assess models in a competing events scenario at the patient level. It is an interevent metric that evaluates the match between the event predicted by the model and the actual event that occurred at a given time point for each patient and takes into account multiple competing events.

In this study, we aimed to develop a MASH cirrhosis–specific deep learning model based on DeepHit using data at the time of waitlisting that accounts for the competing risks of death and transplant on the LT waitlist to forecast waitlist trajectory and inform clinical decision-making. We compared the performance of the DeepHit model to MASH-specific single-risk CoxPH and random survival forest (RSF) models. We externally validated the DeepHit model on single-center data. Finally, we developed a DeepHit dashboard to enter and visualize patient trajectory on the waitlist [21].

## Methods

### Study Design and Participants

We conducted our retrospective study using two study populations. Of 227,647 patients waitlisted in the Scientific Registry of Transplant Recipients (SRTR) from March 1, 2002, to March 2, 2021, we used data from 17,551 patients with MASH cirrhosis for model development (Figure S1). For external validation, we used data from 167 patients with MASH cirrhosis who were waitlisted at University Health Network (UHN), Toronto, Ontario, between 2012 and 2018. Inclusion and exclusion criteria were identical for both cohorts, ensuring consistency in the selection process. We included all adults aged 18 years or older with a primary diagnosis of MASH or cryptogenic cirrhosis (CC) and a BMI of 30 kg/m^2^ or more based on previous studies that demonstrate the histological overlap between MASH cirrhosis and CC [22-24]. We excluded patients with a secondary diagnosis other than MASH cirrhosis or CC with a BMI of 30 kg/m^2^ or more [22,25]. We excluded LT recipients who were never waitlisted, retransplants, multiorgan transplants, acute liver failure (including status 1 and status 1a candidates), concomitant liver etiologies (viral hepatitis B and C and alcoholic liver disease), hepatocellular carcinoma–related primary or secondary diagnosis or listed with exception points, and those with pre-existing liver malignancies; supplementary information 1). Patients were followed for up to 120 months, or until death or deterioration, removal, or transplant, whichever occurred first. Events in both cohorts were classified as (1) death (died on the waitlist or removed due to deterioration), (2) received LT, or (3) censored, and event times were determined accordingly. Candidates removed due to deterioration were included in the death group, while those removed from the waitlist for other reasons were classified as censored (Figure S2). Waitlist outcome categorization is consistent with previous literature [26].

### Models

We developed all models using SRTR data based on shared features between the SRTR and UHN datasets. The DeepHit model includes a shared network with fully connected layers to capture intrinsic patterns of the input features, which are then connected to two cause-specific subnetworks that learn the relationship between the input features, event type, and time of the event [11,27]. DeepHit outputs discrete monthly risk predictions of the competing events. The sum of the monthly prediction over the entire time horizon *T* for all the competing events is 1/*K* , where *K* is the total number of competing events. In our case, the maximum event time is 120 months. The cumulative risk for 1 event over this time period is 0.5, as there are two competing events. The DeepHit model includes a shared network with fully connected layers and 2 cause-specific subnetworks that correspond to death and transplant. The joint distribution of the event and first hitting time is learned and outputted through a final layer to output *r_i_* (*k*, *t*|x_*i*_) defined as


$$r_{i}\left(k,t|x_{i}\right)=f\left(x_{i}\right)$$

which is the predicted probability of the *k* event happening at a specific prediction time *t,* satisfying

$\sum_{k=1,\;t=1}^{K,\;T}r_{i}\left(k,t|x_{i}\right)=1$

where *f* represents the DeepHit structure [11].

**Figure 1.**
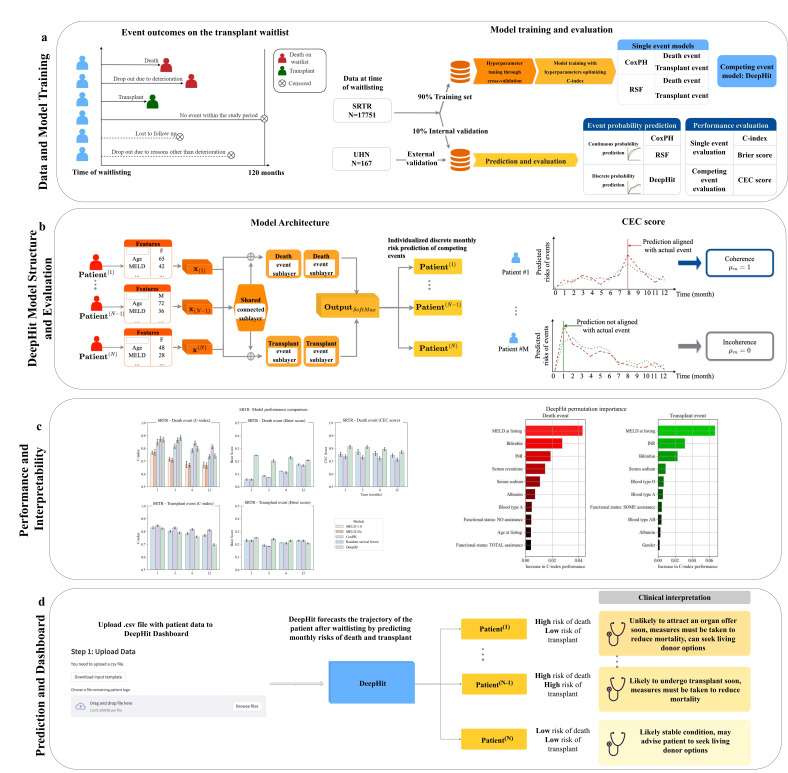
DeepHit model. (A) The model training process, event prediction, and evaluation process are displayed. (B) The DeepHit model architecture is described. (C) The competing event coherence score evaluates model performance based on the prediction of an event compared to the actual event that occurred at the patient level. (D) DeepHit can be used to predict death and transplant and inform clinical decisions. C-index: concordance index; CEC: competing event coherence; CoxPH: Cox proportional hazards; INR: international normalized ratio; MASH: metabolic dysfunction–associated steatohepatitis; MELD: Model for End-Stage Liver Disease; RSF: random survival forest; SRTR: Scientific Registry of Transplant Recipients; and UHN: University Health Network.

We compared DeepHit to single-risk CoxPH and RSF models predicting death or transplant. CoxPH is a linear model that predicts the hazard function with an assumption of proportionality where the individual hazard is proportional to the population baseline hazard that changes over time [14]. (Figure 1) For each cause-specific CoxPH or RSF model predicting death or transplant, the competing event (death or transplant) was censored. Model hyperparameters are available in Table S1 in Multimedia Appendix 1. For comparison, MELD-Na and MELD 3.0 scores were calculated to predict mortality (supplementary information 1)

### Model Evaluation

Models were evaluated using the C-index and Brier scores. Furthermore, we propose a new metric to evaluate the percentage of prediction that is coherent to the actual event type and time at which it occurred in competing risk scenarios, known as the CEC score (or the *µ*-score). This overcomes the limitations of the C-index and the Brier score, which examine model performance under a single-risk scenario but cannot be used to compare all the predicted risks for the competing events at the same time. Ideally, at the time of the actual event that occurred, the predicted risk for that event should be higher than the predicted risk of other competing events. For the patients who had an event (death or transplant) within the time frame of interest, the percentage of coherence within the population was calculated. The CEC score measures the alignment between the model risk predictions and the actual patient event that occurred. For the *M* patients who had the event within the time frame *t*, the percentage of coherence within the population was calculated. The CEC score measures the alignment between the risk predictions and the actual patient event. Let’s *µ*_*m*_ indicate the coherence status of the $m^{th}$ patient being evaluated. Further, let l* and k*_m_ denote the actual event time and event type of the $m^{th}$ patient, respectively; then the proposed *µ*-score is defined as


$$\mu -score=\frac{1}{M}\Sigma_{m=1}^{M}\mu_{m}$$

where coherence for the *m* patient *μ*_*m*_ is defined as


$$\mu_{m}=\left\{ \begin{array}{ll} 1, & \text{if }\arg\max_{k\in K}r_{m}(k,\ell^{*}|x_{m})=k_{m}^{*}\\ 0, & \text{otherwise}, \end{array}\right.$$

and *r_m_* (*k*, l*|x*_m_*) denotes the predicted risks of the 2 events at the event time. Because CoxPH and RSF are single-risk models, we say that the prediction is in coherence (or alignment) if the probability of experiencing an event Pr_event_ is higher for the event corresponding to the actual event (at time l* as compared to the competing one.

C-index, Brier scores, and CEC scores were computed for all models at four time points after waitlisting: 1, 3, 6, and 12 months. One month corresponds to the 25th percentile of event time in the population [17]. Mortality predictions were also generated using MELD-Na and MELD-3.0 scores for performance comparison. Transplant predictions were not developed for the MELD models since they are only intended to predict death on the waitlist. Since DeepHit is a competing risk model, when the performance was evaluated using single event metrics (C-index and Brier score), the competing event was treated as censored [11].

### Statistical Analysis

To ensure the robustness of our model, we used rigorous cross-validation, including k-fold cross-validation and hyperparameter tuning to optimize model performance and ensure generalizability (supplementary information 2). SRTR data were split using stratified random split to preserve the event rate of the population in the training and test sets, which represented 90% and 10% of the entire population, respectively. Within the 90% training set, outer five-fold cross-validation was used to obtain an average performance across all validation folds.

Hyperparameter tuning was used to optimize the C-index. To evaluate the performance of our trained models, we first used single-risk metrics, including the time-dependent C-index and the time-dependent Brier score. The model with the highest performance on the outer validation set was used to test performance on the 10% test set as well as the UHN external validation set. Test performance was evaluated at 1, 3, 6, and 12 months using bootstrapping to obtain consistent results where each bootstrapped sample contains patients that were randomly sampled from the cohort with replacement. The Wilson Cox test was subsequently used to test statistical significance in the performance of DeepHit and the other models (CoxPH and RSF) based on the bootstrapped C-indices, Brier scores, and CEC score proposed as the following (Table S1).

### Model Interpretability

Permutation importance was used to determine which covariates in the DeepHit model have the largest influence on the prediction of death and transplant by evaluating the contribution of each covariate to the C-index of the model via random permutation of each variable, which is then compared to the original data [28]. The permutation was done 20 times for each variable to obtain the average and SD of increase in C-index.

All analyses were done using Python version 3.8.8 (Python Software Foundation). CoxPH models were developed using the scikit-learn 1.1.1 library and the scikit-survival 0.16.0 library. The MASH-specific DeepHit model was developed using the TensorFlow 0.0.8 library. The data template can be downloaded as a .csv file. The codebase has been published on GitHub [29].

### Ethical Considerations

Due to the use of publicly available deidentified United Network for Organ Sharing (UNOS) data, this study was exempt from Research Ethics Board (REB) review. For external validation, REB approval was obtained (REB number 21‐5783).

## Results

### Characteristics of SRTR and UHN Cohorts

There were 17,551 patients with MASH in the SRTR cohort, of which 50.2% (8802/17551) were female. Mean MELD at listing was 19.4 (SD 8.1). By 120-month waitlist follow-up, 54.6% (9599/17551) of patients underwent LT, 25.6% (4510/17551) of patients died or were removed from the waitlist due to deterioration, and 19.8% (3442/17551) of patients were removed for improvement or were censored. Around 93.8% (9004/9599) of recipients underwent deceased donor liver transplantation (DDLT). There were 167 patients with MASH cirrhosis in the UHN dataset, and 46.1% (77/167) were female. The mean MELD at listing was 22.1 (SD 6.6). Overall, 62.9% (105/167) of patients underwent LT, of which 72.4% (76/105) of patients underwent DDLT, 23.4% (39/167) died, and 13.7% (23/167) were removed or censored (see supplementary information 1 and Table S2 in Multimedia Appendix 1). Additional features in the SRTR and UHN datasets are available in Table S3 in Multimedia Appendix 1.

### Model Performance

**Figure 2.**
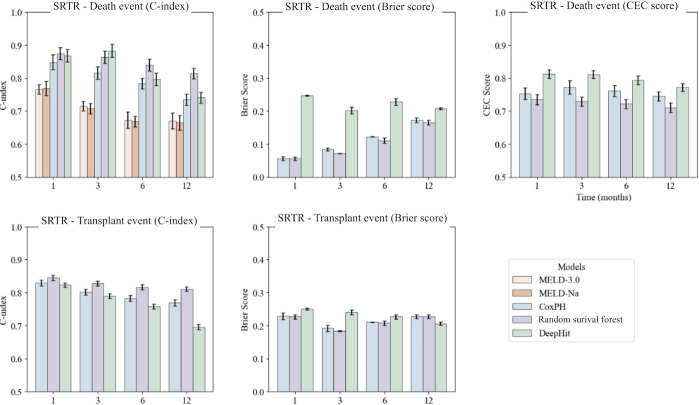
Model performance comparison with the Scientific Registry of Transplant Recipients data*.* Performance of models evaluated using C-index, Brier score, and CEC score on SRTR data. CEC: competing event coherence; C-index: concordance index; CoxPH: Cox proportional hazards; MELD: Model for end-stage liver disease; SRTR: Scientific Registry of Transplant Recipients.

In the competing risk scenario, DeepHit consistently achieved statistically significant higher CEC scores at each time point evaluated on the SRTR and UHN datasets (Figures2  3). Numerical results are displayed in supplementary information 6. When comparing model performance using SRTR data (Figure 2), CoxPH, RFS, and DeepHit had higher C-indices than MELD-Na and MELD 3.0 for death events at 1, 3, 6, and 12 months, outperforming MELD-Na and MELD 3.0 at all time points. RSF achieved statistically significantly higher C-indices (*P*<.01) except in the RSF-DeepHit comparison at 3 months for death event and 1 month for transplant. RSF had statistically significantly lower Brier scores at each time point evaluated for death and transplant, except for at 12 months for the transplant event (DeepHit: 0.206 vs RSF: 0.228).

**Figure 3.**
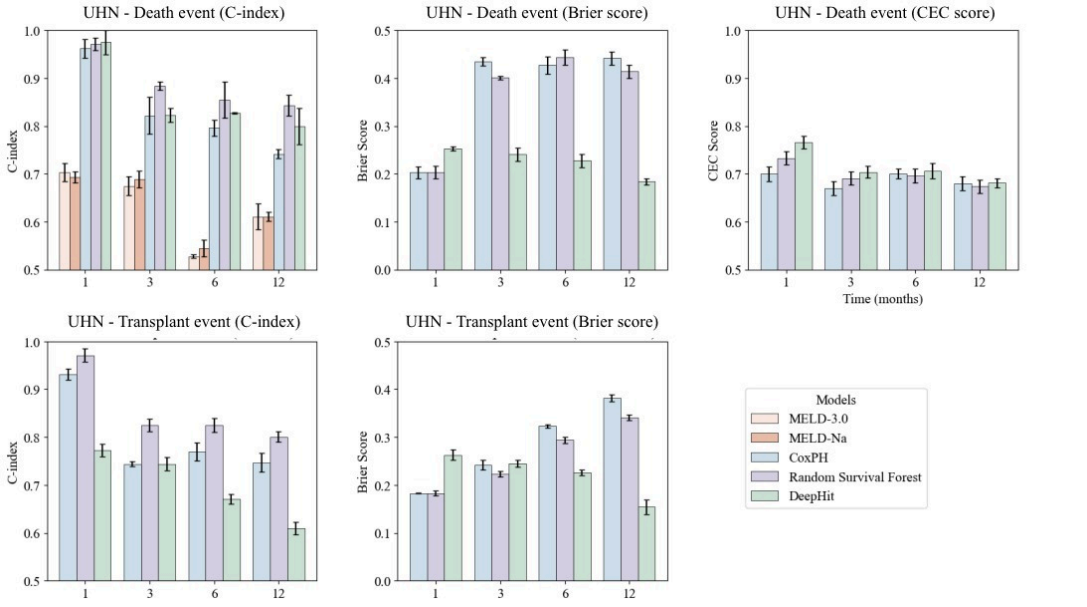
Model performance comparison and external validation of the model using University Health Network data. Performance of models evaluated using C-index, Brier score, and CEC score on UHN data. CEC: competing event coherence; C-index: concordance index; CoxPH: Cox proportional hazards; MELD: Model for end-stage liver disease; SRTR: Scientific Registry of Transplant Recipients.

In external validation, DeepHit demonstrated a statistically significantly higher C-index at 1 month for death (0.975); however, RSF had the best performance at all other time points for the death and transplant events. DeepHit also exhibited lower Brier scores at 3, 6, and 12 months compared to RSF for death. For the transplant event, RSF had lower Brier scores at 1 and 3 months than DeepHit, while CoxPH and RSF performed better at 6 and 12 months (Figure 3). Full numerical results can be found in Table S4 in Multimedia Appendix 1.

### Forecasting Waitlist Trajectory With DeepHit

**Figure 4.**
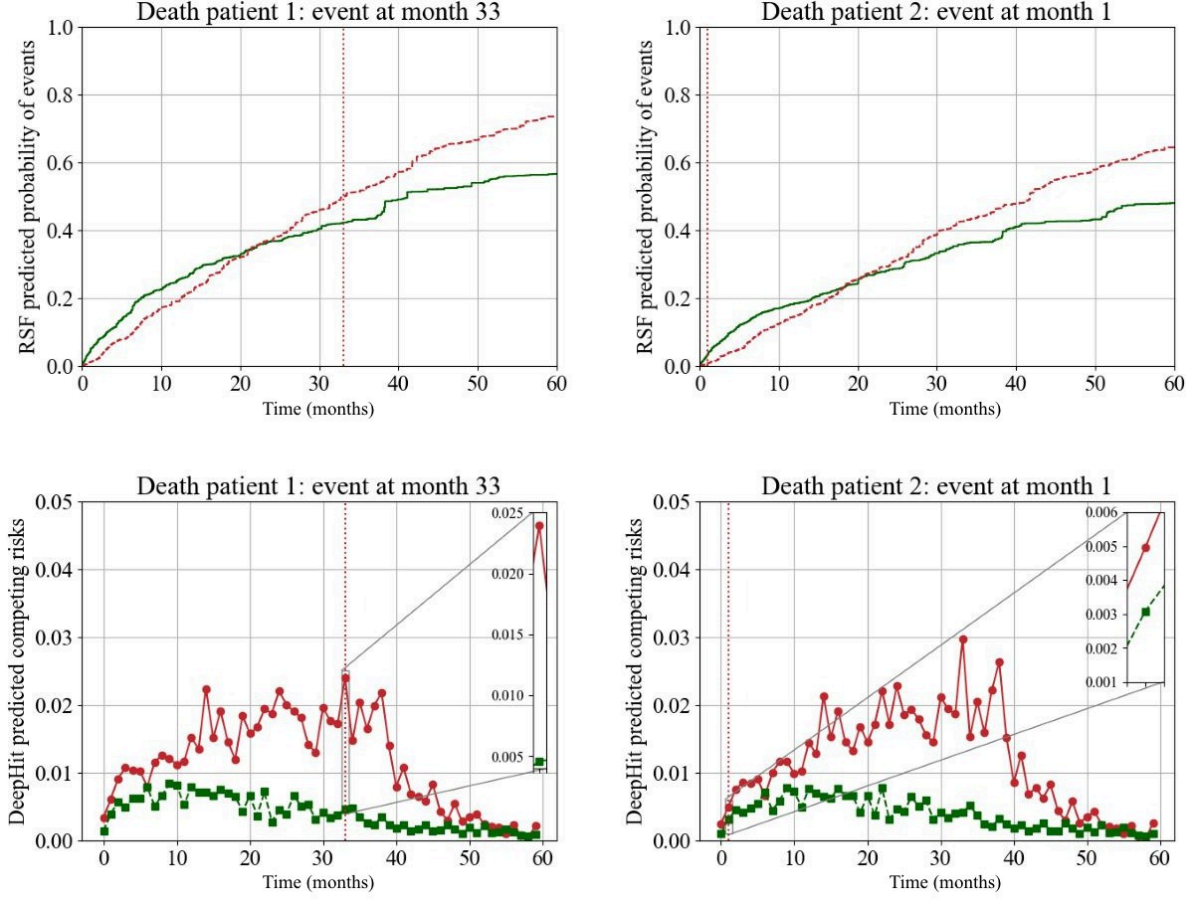
Forecasting death on the waitlist using Random Survival Forest and DeepHit with patient examples using data at the time of waitlisting. The vertical line on each plot indicates the event (green=transplant; red=death) and the time at which it occurred. On the DeepHit plots, zoomed-in segments correspond to the time at which the actual event occurred. For a patient that experienced an event during month 1, they were categorized as experiencing the event at time 0. For a patient that experienced an event during months 1 and 2, they were categorized as experiencing the event at month 1 and so on.

**Figure 5.**
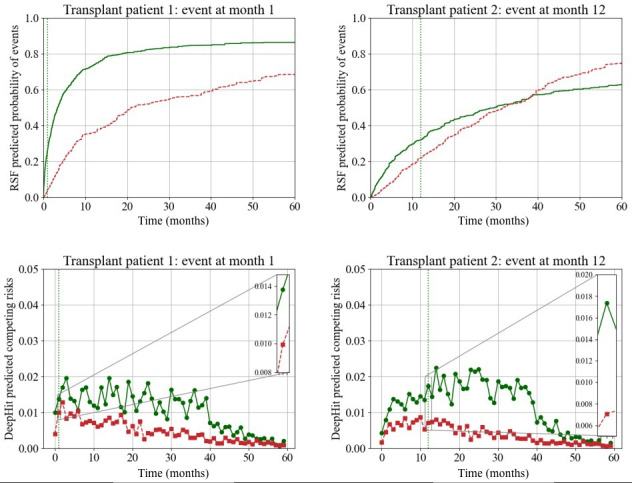
Forecasting transplant on the waitlist using random survival forest and DeepHit. Similar to Figure 4, transplant on the waitlist is predicted using RSF and DeepHit with each plot corresponding to a different patient.

Four patients from the SRTR were randomly selected, and their waitlist trajectories of death and transplant were predicted using RSF and DeepHit over a 120-month period based on data available at the time of waitlisting. Figure 4 displays two patients who died on the waitlist at month 33 and month 1, while Figure 5 displays two patients who were transplanted at month 1 and 12. The time at which the event (death or transplant) occurred and the corresponding prediction at that time is magnified. Patient characteristics for the sample patients used here are detailed in Table S5 in Multimedia Appendix 1. Cumulative risk predictions with RSF were generated using two separate models with death or transplant as the outcome of interest and displayed on one graph. Using DeepHit, we generated granular risk predictions from one competing risk model, allowing for visualization of the risk associated with each event over time compared.

### Permutation Importance Using DeepHit

**Figure 6.**
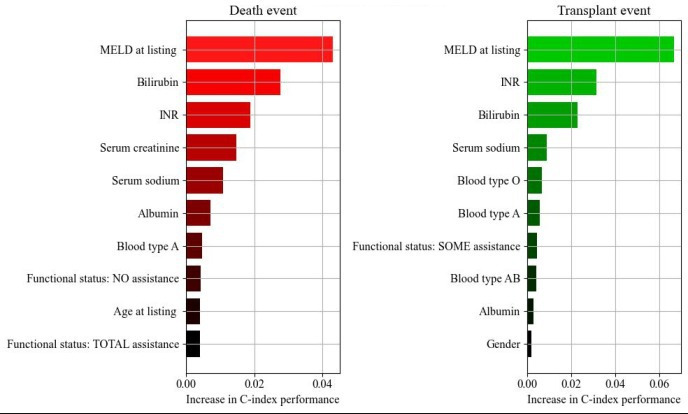
Permutation importance of DeepHit model. Features with the greatest contribution to the prediction of death or transplant were determined using permutation importance. INR: international normalized ratio; MELD: Model for end-stage liver disease

In predicting the death event, MELD at listing emerged as the greatest contributor to the C-index, followed by bilirubin, INR, serum creatinine, and serum sodium. Additional non-MELD features identified were albumin, blood type A, functional status, and age at listing. For transplant events, MELD at listing took precedence, followed by INR and bilirubin. The non-MELD features that influenced transplantation comprised blood types O, A, and AB, as well as functional status, albumin, and gender (Figure 6).

## Discussion

### Principal Findings

This study aimed to forecast the LT waitlist trajectories of patients with MASH cirrhosis using a deep learning approach that leverages the changing relationships between covariates and risks over time while handling competing risks [11]. We compared a competing risk MASH cirrhosis–specific DeepHit model to single-risk CoxPH and RSF models in forecasting outcomes of death and transplant on the waitlist. We demonstrated that while RSF performed better at most evaluation time points using traditional model performance metrics, DeepHit outperforms RSF when evaluated in a competing risk scenario. Furthermore, we demonstrated that DeepHit can generate and visualize time-varying, discrete predictions of death and transplant. This allows for the identification of patients with a low probability of undergoing transplantation and those at an elevated risk of death while awaiting DDLT, enabling timely consideration of LDLT when available.

MELD-based models poorly predict death in patients with MASH compared to candidates listed for other etiologies and may underestimate the risk of death on the waitlist [5,6]. Additionally, MELD-based models do not provide information on the risk of transplantation. Patients with MASH have a lower 90-day and 1-year probability of transplant and a greater risk of waitlist mortality. Therefore, early identification of patients at low risk of transplant may expedite direction to LDLT and prevent MASH-related mortality on the waitlist [30]. Previous studies have indicated that LDLT results in comparable or improved postoperative outcomes for patients with and without MASH when compared to DDLT. Timely identification of patients enables them to undergo transplantation while in a less decompensated state, leading to more efficient resource usage [31-33]. While MASH-specific models, such as the DeepHit-based model, may not fully replace MELD-based models for waitlist prioritization and liver allocation of all candidates, they can be used to improve the management of waitlisted patients with MASH, particularly those with indolent disease who may spend months on the waitlist and deteriorate before receiving an LT offer. As the prevalence of MASH cirrhosis grows, careful management of waitlisted patients is critical for reducing overall waitlist morbidity and mortality [30]. The DeepHit MASH-specific model also highlights the potential to incorporate personalized prediction of waitlist outcomes at the patient level. This prediction can be updated as clinical status changes over time when modifiable factors associated with a higher risk of waitlist mortality are addressed, providing a more granular prediction of both death and transplant over time on the waitlist as opposed to risk of 90-day mortality alone.

We presented the CEC score as an alternative metric to evaluate competing risk models, emphasizing its role as an interevent measure that assesses a model’s performance in predicting 2 competing events [34]. Unlike the C-index, which evaluates performance at the population level, the CEC score assesses performance at the patient level [12,20]. A major problem with the C-index in the setting of competing risks is that the discrimination of a model is reduced when covariates may be associated with both the primary and competing event [20]. Our analysis demonstrates that DeepHit more accurately predicts the actual event (death or transplant) that occurred at specific time points when evaluated using the CEC score compared to RSF, although RSF performs better based on the C-index and Brier scores. Although the C-index and Brier scores are limited in the scenario of competing risks, DeepHit still exhibited robust performance when evaluated using these metrics, although it was statistically weaker than RSF.

While RSF performed well in a single-risk scenario when evaluated with the C-index and Brier scores, using a noncompeting risk model to predict risk in a clinical setting where patients are at risk of multiple competing events may lead to greater misclassification of risk for single events. We demonstrated this through the improved performance of DeepHit compared to RSF when evaluated with the CEC score. Competing risk analysis may improve predictions of outcomes at the patient level [35-40]. Furthermore, censoring of competing risks can lead to event overestimation in populations at highest risk of experiencing either of the competing events [41]. Competing risk models to evaluate the risks of death and transplant on the waitlist are limited in the field of LT but have been more extensively described and applied to kidney transplantation. Smits et al [42] found that Kaplan-Meier overestimates the chance of transplantation compared to competing risk analysis by over 30% [43]. This not only highlights the importance of competing risk models but also using metrics, such as the CEC score, in the analysis of models to accurately assess performance. We highlight the advantages of DeepHit, whose neural network architecture is particularly well-suited to capturing complex, nonlinear, and interdependent relationships among variables in medium-sized datasets. In contrast to traditional survival models, DeepHit does not rely on restrictive assumptions, such as proportional hazards, enabling greater flexibility in modeling intricate data patterns. Crucially, DeepHit is inherently designed to handle competing risks, estimating the joint probability distribution over multiple, mutually exclusive event types. This multitask learning framework allows the model to share information across outcomes while retaining event-specific distinctions, resulting in more accurate and clinically meaningful survival estimates. By applying a competing risk-specific metric—the CEC score—we demonstrated that DeepHit consistently outperforms traditional ML approaches in predicting time-to-event outcomes with competing risks. These capabilities make DeepHit especially valuable in clinical settings characterized by multiple potential outcomes, such as the organ transplant waitlist.

Previous studies have assessed features associated with death and transplant in LT candidates waitlisted for all indications [5,44-46]. Despite patients with MASH cirrhosis constituting a large and increasing portion of the LT waitlist, limited studies have assessed features associated with death and transplant exclusively in this population [47]. Studies have shown that factors, such as dialysis, sex, race, serum albumin and creatinine, low performance status, and high MELD are associated with increased or decreased risk of transplant among all candidates [5,44-46]. We evaluated the contribution of various covariates in predicting death and transplant events using DeepHit permutation importance, aiming to improve the C-index. In our DeepHit model, MELD at listing emerged as the highest-ranked feature for both death and transplant, followed by components of the MELD score, such as INR, bilirubin, and serum sodium, which have been previously demonstrated [10]. Our permutation analysis reinforced the critical role of MELD-based models in predicting outcomes, showing that biochemical features already included in MELD-based models are implicated in waitlist risk of death and transplant in patients with MASH cirrhosis, potentially reflecting part of the comorbidity burden in this population. Given that SRTR is a historical dataset where allocation is largely determined by MELD score, these findings are not unexpected. Functional status was a highly ranked feature for both events and has been associated with an increased likelihood of transplantation as well as increased waitlist mortality [48]. Poor functional status contributed to the death prediction, possibly because complications of cirrhosis (such as encephalopathy) are associated with poor functional status and increase the risk of death in all LT waitlist candidates [44]. While INR and bilirubin also ranked high, we did not identify creatinine or dialysis in the last week as significant features associated with the prediction of waitlist death or transplant. Age contributed to the death prediction, which has not been found previously in studies on all LT waitlist candidates. Patients with MASH are older, and older patients tend to have poorer waitlist outcomes; therefore, this finding is expected in a MASH cirrhosis cohort [45]. For the transplant event, we found that blood type AB ranked high. These candidates can accept donor livers from nearly all blood types. In other studies, dialysis, sex, race, serum albumin and creatinine, low performance status, and high MELD have been found to be associated with increased or decreased risk of transplant in all candidates [5].

### Limitations

The SRTR is a retrospective dataset and limitations, such as high missingness and heterogeneity of data collection must be noted. Furthermore, there are limited longitudinal features in the SRTR dataset, which makes the development of a dynamic model challenging [49]. The permutation importance method is limited as it does not provide information on the directionality of risk such as provided by a hazard ratio, which makes it difficult to apply DeepHit to clinical scenarios where risk factors can be identified and modified to improve outcomes. Furthermore, none of the models developed consider individual organ availability, donor compatibility, and regional variation. Although the DeepHit model performed well when externally validated on the UHN dataset, the UHN sample size was relatively small at 167 patients. Further work should seek to validate this model on larger datasets. Finally, the use of historical waitlist candidate data to devise a ranking system may perpetuate existing inequities and biases in liver allocation; therefore, DeepHit should not be used for definitive decision-making on waitlisting or delisting but can be used in conjunction with other tools.

### Conclusion

The DeepHit model can be used to forecast waitlist trajectories of both death and transplant in a competing risk scenario for patients with MASH, using data available at the time of listing. With DeepHit, discretized and dynamic changes of the risk of death and transplant can be visualized and compared across multiple time points and between events. This information can enhance the current strategies for managing candidates with LT with MASH cirrhosis and act as a tool to advocate for LDLT in patients who are at high risk of waitlist dropout but underserved by MELD-based mortality predictions. A DeepHit MASH-specific model can be used as a clinical adjunct to inform and modify clinical interventions to optimize patient survival on the waitlist. Future studies should focus on improving the evaluation and interpretability of DeepHit models to expand their use in clinical settings, as well as developing larger scale ML models that consider all patients on the LT waitlist.

## Supplementary material

10.2196/68247 Multimedia Appendix 1 Supplementary figures and tables.

## Abbreviations

C-index: concordance index
CC: cryptogenic cirrhosis
CEC: competing event coherence
CoxPH: Cox proportional hazards
DDLT: deceased donor liver transplantation
INR: international normalized ratio
LDLT: Living donor liver transplantation
LT: liver transplantation
MASH: Metabolic dysfunction-associated steatohepatitis
MELD: Model for end-stage liver disease
MELD-Na: model of end-stage liver disease-sodium
ML: machine learning
REB: Research Ethics Board
RSF: random survival forest
SRTR: Scientific Registry of Transplant Recipients
UHN: University Health Network
UNOS: United Network for Organ Sharing
