# Supplementary material for: Forecasting Waitlist Trajectories for Patients With Metabolic Dysfunction–Associated Steatohepatitis Cirrhosis: A Neural Network Competing Risk Analysis
Source: J Med Internet Res. 2026 Jan 29;28:e68247. doi: 10.2196/68247 (PMC12854276; doi:10.2196/68247)
Supplement: Multimedia Appendix 1 [file jmir-v28-e68247-s001.docx]

**Forecasting Waitlist Trajectories for Patients with Metabolic Dysfunction-Associated Steatohepatitis Cirrhosis Using Neural Network Competing Risk Analysis**

**Figure S1: Patient selection flowchart**


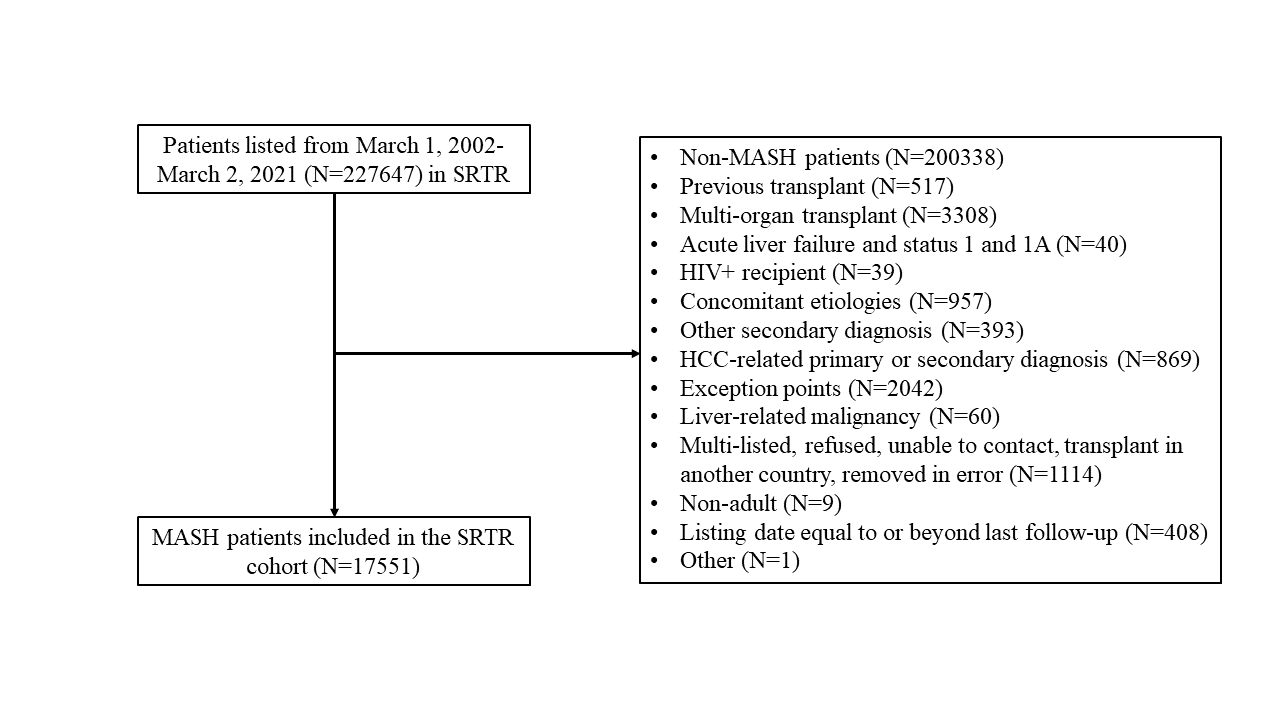


**Figure S2: Classification of waitlist outcomes**


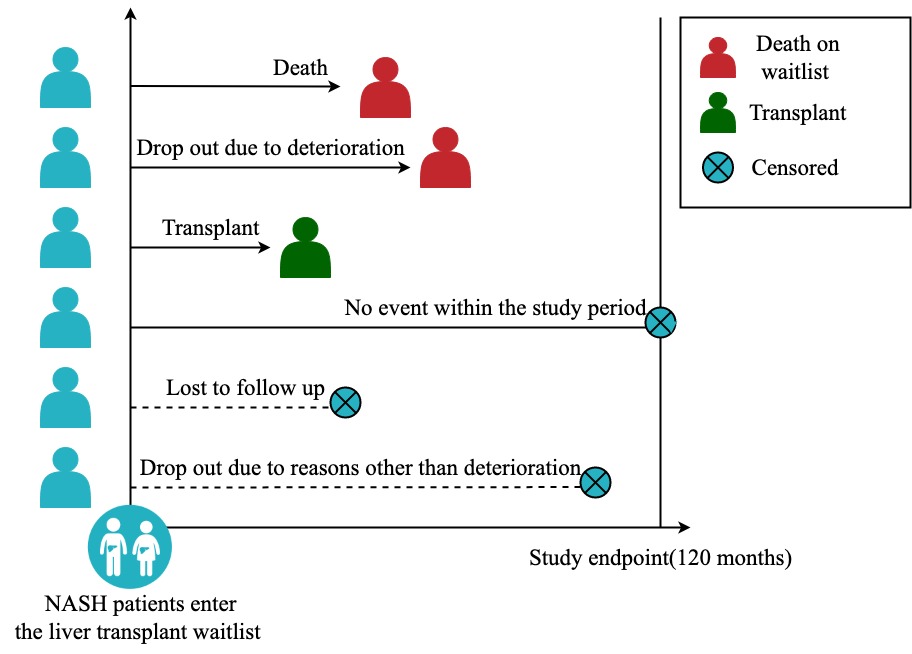


**Table S1: Model parameters and search space.**

| **Models** | **Hyperparameters** | **Search space** |
| --- | --- | --- |
| **RSF** | Maximum features per leaf | {2,3,4} |
|  | Minimum features per leaf | {2,5,10,20,30} |
| **DeepHit** | Batch size | {32,64,128} |
|  | Number of layers | {1,2,3,4,5} |
|  | Nodes | {10,20,30} |
|  | Activation function | {relu,elu,tanh} |
|  | Alpha | {0.1, 0.25, 0.5, 1.0, 2.0, 3.0, 5.0} |
|  | Beta | {0.1, 0.25, 0.5, 1.0, 2.0, 3.0, 5.0} |
|  | Gamma | {0.1, 0.25, 0.5, 1.0, 2.0, 3.0, 5.0} |

**Supplementary Information 1: Data processing and model training process**

All variables used were LT candidate covariates that were available in the SRTR dataset and measured at the time of waitlisting. One-hot encoding was used to create dummy variables for categorical variables. For simplicity, various SRTR variable levels were combined. Three employment status variables in the SRTR dataset (can_empl_stat, can_work_yes_stat, and can_work_no_stat) were combined into one variable with five options: full-time, part-time, work limited by disease, not working, and unknown. Functional status (can_functn_stat) was divided into three categories: total assistance (≤50%), some assistance (60-80%), and no assistance (90-100%). Presence of peptic ulcer disease (can_peptic_ulcer) was split into a binary variable. Payment variables (can_primary_pay and can_secondary_pay) were split up into the following categories: private insurance, public insurance, self-pay, donation, free, pending, foreign government, and unknown. Binary variables with value ‘Unknown’ were replaced with numerical value 0.5, as the average between 1 for ‘Yes’ and 0 for ‘No’. Mean imputation was applied to missing entries in the continuous variables in the data.

For all models, 90% and 10% of data was reserved for training and testing, respectively. Within the 90% training set, outer five-fold cross-validation was used to obtain an average performance of across all validation folds. The model with the highest performance on the outer validation set was used to test performance on the 10% test set. Within each fold in the outer cross-validation process, inner five-fold cross-validation was used to tune hyperparameters that give the highest C-index in the inner validation set.

For the CoxPH model, an elastic net with an $\mathcal{l}_{1} \text{to }\mathcal{l}_{2}$ ratio of 0.9 was chosen to regularize the coefficients of the covariates to prevent overfitting and achieve feature selection. Penalty strength alpha was optimized where the alpha value that maximized concordance-index (C-index) performance was selected. Similarly, the maximum features per leaf and minimum samples per leaf for the RSF model were optimized using the same method. The hyperparameters and their search space are detailed in supplementary table 1.

For the DeepHit model, the joint distribution of the event and first hitting time is learnt and outputted through a final layer to output $r_{i}\left( k,t | \mathbf{x}_{i} \right)$ defined as

$$r_{i}\left( k,t | \mathbf{x}_{i} \right)=f\left( \mathbf{x}_{i} \right)$$

which is the predicted probability of the $k$ event happening at a specific prediction time $t$, satisfying

$$\sum_{k=1, t=1}^{K, T} r_{i}\left( k,t | \mathbf{x}_{i} \right)=1,$$

where $f$ represents the DeepHit structure.^1^

In the model training and tuning process for DeepHit, the learning rate was set as $1e^{-4}$ and the dropout rate as 0.4. Five outer iterations of random search were conducted where a set of hyperparameters was selected at each iteration. Within each outer iteration, 50000 training iterations were done. The optimal combination of hyperparameters that maximized the C-index on the validation set was selected. The search space is summarized in Table 1.

**Table S2: Waitlist outcomes in SRTR and UHN dataset.**

| **Outcomes** | **SRTR (n (%))** | **UHN (n (%))** |
| --- | --- | --- |
| **Overall** | | |
| **Transplanted: n (%)** | 9599 (54.6) | 105 (62.9) |
| **Died* n (%)** | 4510 (25.6) | 39 (23.4) |
| **Removed/Censored** n (%)** | 3442 (19.8) | 23 (13.7) |
| **MELD at listing: mean (SD)** | 19.4 (8.1) | 22.1 (6.6) |
| **Transplanted Candidates: mean (SD)** | | |
| **MELD at listing** | 16.8 (7.7) | 22.5 (6.3) |
| **MELD at transplant** | 23.9 (8.5) | 24.9 (7.4) |
| **Mean time to transplant, months** | 6.2 (11.2) | 6.2 (8.6) |
| **Deceased donor: n (%)** | 9004 (93.8) | 76 (72.4) |
| **Died on waitlist: mean (SD)** | | |
| **MELD at listing** | 21.2(8.0) | 23.9 (7.5) |
| **MELD at death** | 21.1 (10.2) | 26.6 (9.1) |
| **MELD at removal** | 21.8 (10.5) | 16.9 (4.6) |
| **Mean time to death, months** | 16.9 (7.7) | 7.2 (8.8) |

***** Including removed condition deteriorated/too sick

**Including removed conditioned improved

**Table S3: Shared features between SRTR and UHN used for model development (**N=No, Y=Yes, U=Unknown)

| **Variable (SRTR code)** | **SRTR Missingness** | **UHN**  **Missingness** | **Category** | **SRTR** | **UHN** |
| --- | --- | --- | --- | --- | --- |
|  |  |  |  |  |  |
| **Gender, n (%)**  (CAN_GENDER) | 0 | 0 | Female | 8802 (50.2) | 77 (46.1) |
|  |  |  | Male | 8749 (49.8) | 90 (53.9) |
| **Age at waitlisting, mean (SD)**  (CAN_AGE_AT_LISTING) | 0 | 0 |  | 58.5 (8.4) | 59.9 (7.2) |
| **Blood type, n (%)**  (CAN_ABO) | 0 | 0 | A | 6750 (38.2) | 66 (39.5) |
|  |  |  | B | 1966 (11.1) | 9 (5.4) |
|  |  |  | AB | 677 (4.2) | 23 (13.8) |
|  |  |  | O | 8158 (46.5) | 69 (41.3) |
| **BMI, mean (SD)**  (CAN_BMI) | 40 (0.2) |  |  | 33.1 (6.2) | 31.5 (6.2) |
| **Ascites, n (%)**  (CAN_ASCITES) | 16896 (96.3) | 0 | N | 110 (16.8) | 12 (7.2) |
|  |  |  | U | 16 (2.4) | - |
|  |  |  | Y | 529 (80.8) | 155 (92.8) |
| **Spontaneous Bacterial Peritonitis, n (%)**  (CAN_BACTERIA_PERIT) | 0 | 2 (1.2) | N | 15984 (91.1) | 121 (73.3) |
|  |  |  | U | 207 (1.2) | - |
|  |  |  | Y | 1360(7.7) | 44 (26.7) |
| **Diabetes, n (%)**  (CAN_DIAB) | 16896 (96.3) | 0 | No | 355 (2.0) | 66 (39.5) |
|  |  |  | U | 19609 (96.3) | - |
|  |  |  | Yes | 290 (1.7) | 101 (60.5) |
| **Dialysis, n (%)**  (CAN_DIAL) | 10086 (57.5) | 0 | N | 6969 (93.4) | 149 (89.2) |
|  |  |  | U | 57(0.7) | - |
|  |  |  | Y | 439(5.9) | 18 (10.8) |
| **Drug-treated hypertension, n (%)**  (CAN_DRUG_TREAT_HYPERTEN) | 10243 (58.3) | 0 | N | 4511 (61.7) | 93 (55.7) |
|  |  |  | U | 130 (1.8) | - |
|  |  |  | Y | 2667 (36.5) | 74 (44.3) |
| **Encephalopathy**  (CAN_ENCEPH) | 16896 (96.3) | 0 | N | 222 (33.9) | 32 (19.2) |
|  |  |  | U | 15 (2.3) | - |
|  |  |  | Y | 418 (63.8) | 135 (80.8) |
| **Functional status**  (CAN_FUNC_STAT) | 661  (3.77) | 0 | Total | 1907 (11.3) | 1 (0.7) |
|  |  |  | Some | 9272 (54.9) | 109 (71.7) |
|  |  |  | None | 5711 (33.8) | 42 (27.6) |
| **Medical Condition**  (CAN_MED_COND) |  |  | U | 9831 (56.0) | 2 (1.2) |
|  |  |  | Not hospitalized | 407 (2.3) | 28 (16.8) |
|  |  |  | Hospitalized | 7313 (41.7) | 137 (82.0) |
| **Variceal Bleeding**  (CAN_VARICEAL_BLEEDING) | 10228 (58.3) | 0 | N | 6800 (92.9) | 52(31.1) |
|  |  |  | U | 184 (2.5) | - |
|  |  |  | Y | 339 (4.6) | 115(68.9) |
| **Laboratory Variables: mean (SD)** |  | | |  |  |
| **MELD at listing**  (CAN_INIT_ACT_STAT_CD) | 0 | 0 |  | 19.4 (8.1) | 22.1 (6.6) |
| **Serum albumin**  (CANHX_ALBUMIN) | 0 | 0 |  | 30.6(6.3) | 30.9 (5.5) |
| **Serum Creatinine**  (CANHX_SERUM_CREAT) | 0 | 0 |  | 126.2 (98.3) | 124.0 (82.6) |
| **Total Serum Bilirubin**  (CANHX_BILI) | 0 | 0 |  | 89.2 (129.0) | 86.0 (130.6) |
| **Serum sodium**  (CANHX_SERUM_SODIUM) | 0 | 0 |  | 135.9(4.7) | 133.9 (4.9) |
| **INR**  (CANHX_INR) |  | 0 |  | 1.7 (0.6) | 1.7 (0.5) |

**Table S4: Model performance results.**

| Model | Time (months) | SRTR | | | | | UHN | | | | |
| --- | --- | --- | --- | --- | --- | --- | --- | --- | --- | --- | --- |
|  |  | Death | | Transplant | | CEC | Death | | Transplant | | CEC |
|  |  | C-index | Brier | C-index | Brier |  | C-index | Brier | C-index | Brier |  |
| CoxPH | 1 | 0.848 | 0.056 | 0.829 | 0.229 | 0.753 | 0.962 | **0.203** | 0.931 | 0.183 | 0.7 |
| RSF | 1 | **0.874** | **0.056** | 0.845 | **0.227** | 0.735 | 0.971 | 0.204 | **0.971** | **0.183** | 0.733 |
| DeepHit | 1 | 0.868 | 0.247 | 0.823 | 0.251 | **0.813** | **0.975** | 0.253 | 0.772 | 0.263 | **0.766** |
| CoxPH | 3 | 0.816 | 0.084 | 0.802 | 0.192 | 0.772 | 0.822 | 0.435 | 0.744 | 0.242 | 0.67 |
| RSF | 3 | 0.864 | **0.072** | **0.828** | **0.184** | 0.729 | **0.884** | 0.401 | **0.825** | **0.223** | 0.691 |
| DeepHit | 3 | 0.883 | 0.202 | 0.79 | 0.241 | **0.811** | 0.823 | **0.241** | 0.744 | 0.245 | **0.704** |
| CoxPH | 6 | 0.784 | 0.122 | 0.783 | 0.211 | 0.761 | 0.796 | 0.627 | 0.77 | 0.323 | 0.7 |
| RSF | 6 | **0.84** | **0.111** | **0.817** | **0.208** | 0.722 | **0.855** | 0.594 | **0.825** | 0.294 | 0.696 |
| DeepHit | 6 | 0.796 | 0.228 | 0.758 | 0.227 | **0.794** | 0.827 | **0.228** | 0.671 | **0.226** | **0.707** |
| CoxPH | 12 | 0.735 | 0.173 | 0.769 | 0.227 | 0.745 | 0.742 | 0.842 | 0.747 | 0.382 | 0.68 |
| RSF | 12 | **0.814** | **0.166** | **0.811** | 0.228 | 0.711 | **0.843** | 0.814 | **0.801** | 0.341 | 0.674 |
| DeepHit | 12 | 0.741 | 0.208 | 0.696 | **0.206** | **0.772** | 0.8 | **0.184** | 0.609 | **0.154** | **0.681** |

**Table S5 Characteristics of sample patients used in trajectory prediction with RSF and DeepHit.**

| **Characteristics** | **Patient 1** | **Patient 2** | **Patient 3** | **Patient 4** |
| --- | --- | --- | --- | --- |
| Gender | Female | Female | Male | Male |
| Age | 66 | 55 | 54 | 67 |
| Race | White | White | White | White |
| BMI | 48 | 34 | 32 | 38 |
| Initial MELD | 17 | 38 | 22 | 12 |
| Albumin | 28 | 31 | 28 | 31 |
| Bilirubin | 41.04 | 116.3 | 88.9 | 30.8 |
| Serum Creatinine | 114.9 | 433.4 | 114.9 | 88.4 |
| INR | 1.5 | 2.8 | 1.8 | 1.3 |
| Comorbidities | Previous abdominal surgery, malignancy, hospitalized | Previous transfusion, mechanical  ventilation,  hemodialysis,  drug-treated hypertension | Spontaneous bacterial peritonitis | Previous transfusion |
